# Identification of Candidate Driver Genes in Common Focal Chromosomal Aberrations of Microsatellite Stable Colorectal Cancer

**DOI:** 10.1371/journal.pone.0083859

**Published:** 2013-12-18

**Authors:** George J. Burghel, Wei-Yu Lin, Helen Whitehouse, Ian Brock, David Hammond, Jonathan Bury, Yvonne Stephenson, Rina George, Angela Cox

**Affiliations:** 1 Institute for Cancer Studies, Department of Oncology, Cancer Research UK/Yorkshire Cancer Research Sheffield Cancer Research Centre, University of Sheffield, Sheffield, United Kingdom; 2 Academic Unit of Surgical Oncology, Department of Oncology, Cancer Research UK/Yorkshire Cancer Research Sheffield Cancer Research Centre, University of Sheffield, Sheffield, United Kingdom; 3 Department of Histopathology, Sheffield Teaching Hospitals Foundation Trust, Cancer Research UK/Yorkshire Cancer Research Sheffield Cancer Research Centre, University of Sheffield, Sheffield, United Kingdom; 4 Department of Infection and Immunity, University of Sheffield, Sheffield, United Kingdom; Ohio State University Medical Center, United States of America

## Abstract

Colorectal cancer (CRC) is a leading cause of cancer deaths worldwide. Chromosomal instability (CIN) is a major driving force of microsatellite stable (MSS) sporadic CRC. CIN tumours are characterised by a large number of somatic chromosomal copy number aberrations (SCNA) that frequently affect oncogenes and tumour suppressor genes. The main aim of this work was to identify novel candidate CRC driver genes affected by recurrent and focal SCNA. High resolution genome-wide comparative genome hybridisation (CGH) arrays were used to compare tumour and normal DNA for 53 sporadic CRC cases. Context corrected common aberration (COCA) analysis and custom algorithms identified 64 deletions and 32 gains of focal minimal common regions (FMCR) at high frequency (>10%). Comparison of these FMCR with published genomic profiles from CRC revealed common overlap (42.2% of deletions and 34.4% of copy gains). Pathway analysis showed that apoptosis and p53 signalling pathways were commonly affected by deleted FMCR, and MAPK and potassium channel pathways by gains of FMCR. Candidate tumour suppressor genes in deleted FMCR included *RASSF3*, *IFNAR1*, *IFNAR2 and NFKBIA* and candidate oncogenes in gained FMCR included *PRDM16*, *TNS1, RPA3 and KCNMA1*. In conclusion, this study confirms some previously identified aberrations in MSS CRC and provides *in silico* evidence for some novel candidate driver genes.

## Introduction

Colorectal cancer (CRC) is the third most common cancer in males and the second in females [1]. More than 1 million new CRC cases are diagnosed annually and ~600,000 related deaths were estimated worldwide in the year 2008, making CRC the 3^rd^ highest cause of cancer related death in both genders [1,2]. Chromosomal instability (CIN) is the most common form of genomic instability in CRC and it is associated with 65-85% of sporadic CRC cases [3–6]. Tumours that develop through the CIN pathway are characterised by frequent numerical and/or structural gains and losses of chromosomal segments or whole chromosomes at a significantly increased rate in comparison to normal cells [7]. CIN tumours are known to be associated with *TP53* mutations and low levels of microsatellite instability (MSI) [8,9]. CIN is thought to drive CRC development through copy number gain of oncogenes such as *MYC* and the deletion of tumour suppressor genes such as *SMAD4* and *TP53* [3,9–13]. This view is supported by the association observed between copy number abnormalities of cancer-related genes, and their expression levels in CRC samples [14–16].

Although most of the chromosomal aberrations arise in a random fashion, some are recurrent and are commonly found in other types of cancer in addition to CRC [13,16,17]. The increased frequency of some somatic copy number aberrations (SCNA) is probably a result of clonal selection during tumour development. Recurrent SCNA provide the tumour with a way of targeting tumour suppressor genes and oncogenes to acquire one or more of the cancer hallmarks and drive tumorigenesis [13]. Several common chromosomal abnormalities have been identified through conventional cytogenetic techniques, such as metaphase comparative genome hybridisation (CGH) and fluorescent in situ hybridisation (FISH) [18–20]. These chromosomal defects include gains of 8q, 13q and 20q and losses of 18q, 5q, 8p, 17q [18,20]. However, due to their large size, identification of specific driver genes within these regions is problematic [16,17,21]. 

The use of array-based CGH allows the acquisition of genome-wide information with high resolution (down to a few kilobases) and the identification of focal and minimal common regions (FMCR) [16,17]. FMCR are usually smaller than 3Mb in size and thus contain a relatively small number of genes, hence simplifying the identification of driver genes [16,17]. Recently, FMCR have led to the identification of novel cancer driver genes with potential therapeutic and prognostic value in several cancer types including CRC [16,17,22–27]. The main aim of this work was to apply a number of analytical approaches based on high-resolution array-based CGH data for a set of sporadic microsatellite stable (MSS) CRC tumours to both replicate observations of aberrations identified in previous studies and identify novel candidate CRC driver genes. 

## Materials and Methods

### Ethics Statement

Subjects gave written informed consent for data and sample collection and the study was approved by South Yorkshire Research Ethics Committee (UK) (09/H1310/54).

### Study subjects and DNA samples

Tissue samples were available from 53 patients with MSS colorectal tumours. Thirty-eight of these were undergoing surgery for a primary colorectal tumour at the Sheffield Royal Hallamshire and the Sheffield Northern General hospitals (March, 2001 – June, 2005). Fifteen of the case tissue samples were from Sheffield Royal Hallamshire Hospital tissue bank (HTA License 12182). Before inclusion in the study, the tumour status of all the samples was confirmed by a pathologist (JB). All tumour tissue samples were micro-dissected prior to DNA extraction, such that the extracted material contained at least 80% cancerous cells. DNA samples from peripheral blood or normal colon tissues were also available from all of the recruited patients. Additional data including; gender, tumour location, degree of differentiation, stage and age of diagnosis were available from the pathology records (Materials and Methods S1). Genomic DNA was extracted from the tumour, normal tissue and peripheral blood samples using the QIAamp DNA Minikit, (Quiagen, Hilden, Germany).

### MSI status

The MSI status of all the tumour DNA samples was determined using the MSI Analysis System kit, v1.2 (Promega, Madison, USA), based on the mononucleotide microsatellite markers BAT-25, BAT-26, NR-21, NR-24 and MONO-27. PCR products were separated by capillary electrophoresis using an ABI PRISM^TM^ 3730 DNA Analyser and analysed using genemapper software v4.0 (Applied Biosystems, Warrington, UK). Samples without any unstable markers were classified as MSS [28]. The MSI analysis kit also includes 2 highly polymorphic penta-nucleotide markers that were used to check sample identity.

### Sequencing and mutation analysis

For mutation analysis of *BRAF* exon 15, *KRAS* exon 2, *APC* mutation cluster region (MCR), *TP53* exons 4-9 and *PIK3CA* exons 9 and 12, the respective exons were PCR amplified (Primer sequences listed in Materials and Methods S1), and sequenced using PRISM^TM^ BigDye Terminator v3.1 standard prototcol (Applied Biosystems). Sequence data were analysed using the software STADEN [29] and mutations were confirmed by comparison with the NCBI reference sequence. Accession numbers are provided in Materials and Methods S1.

### aCGH profiling

Agilent whole genome CGH arrays 4x44K (Design ID 014950) and 4x180K (Design ID 022060) were applied on 6 and 47 samples respectively (Agilent Technologies, Santa Clara, CA, USA). The arrays contained 60-mer oligonucleotides probes for 42,494 (44K) and 170,334 (180K) distinct chromosomal locations with median probe spacing of 43kb (44K) and 13kb (180K). Analyses were performed according to Agilent oligonucleotide array-based CGH protocol v6.0. Quality assessment of the arrays was based on the derivative log ratio spread (DLRS), signal intensity and reproducibility, background noise, array grid placement and outlier probes presented by Agilent feature extraction software (v 10.5.1.1) as 11 well defined QC metrics (Microarray data access information: GSE 418413).

### Array CGH data analysis

The aCGH analysis was performed using the Agilent genomic workbench software (v 5.0.14). SCNA were detected using the quality weighted interval score algorithm, also called the aberration detection method 2 (ADM2) algorithm (Threshold: 6.0) with default centralisation and fuzzy zero correction. Default feature and aberration filters were applied and intra-array probe replicates were combined. Tumour samples were considered chromosomally unstable if one or more significant aberrations were identified [8]. Recurrent SCNA were identified using the context corrected common aberration (COCA) algorithm with a chromosomal scope, a p-value of 0.05 and overlap threshold of 9.0.

FMCR were defined as regions that are less than 3Mb in size and defined by at least 2 independent focal or overlapping SCNA (considered as size determining events (SDE)). A minimum frequency of >10% of the cases and a COCA score of ~2.0 (p-value=0.01) were also required. 

### Technical validation

In order to validate the aCGH results, 2 duplicate experiments using 44K and 180K arrays were performed. Moreover, 3 FMCR (2 deleted and 1 amplified) were confirmed using copy number quantitative PCR. *TP53* LOH analysis based on the sequencing results was used to confirm 17p deletions.

## Results

### CIN, MSI and mutation status

The 53 samples selected for this study were MSS. The analysis of *APC, TP53*, *KRAS*, *BRAF* and *PIK3CA* mutations indicated that the frequency and pattern of these mutations agree with previously published data on MSS sporadic CRC [30–33] and (http://www-p53.iarc.fr/index.html). Of the 53 CRC cases successfully analysed by array CGH, 5 had no significant aberrations and were considered chromosomally stable. The ADM2 algorithm failed to call aberrations for 2 samples and a further sample failed on >4 QC metrics. A summary of the molecular features of the 53 samples is presented in Table S1 in File S1.

### Distribution of Common SCNA

**Figure 1:**
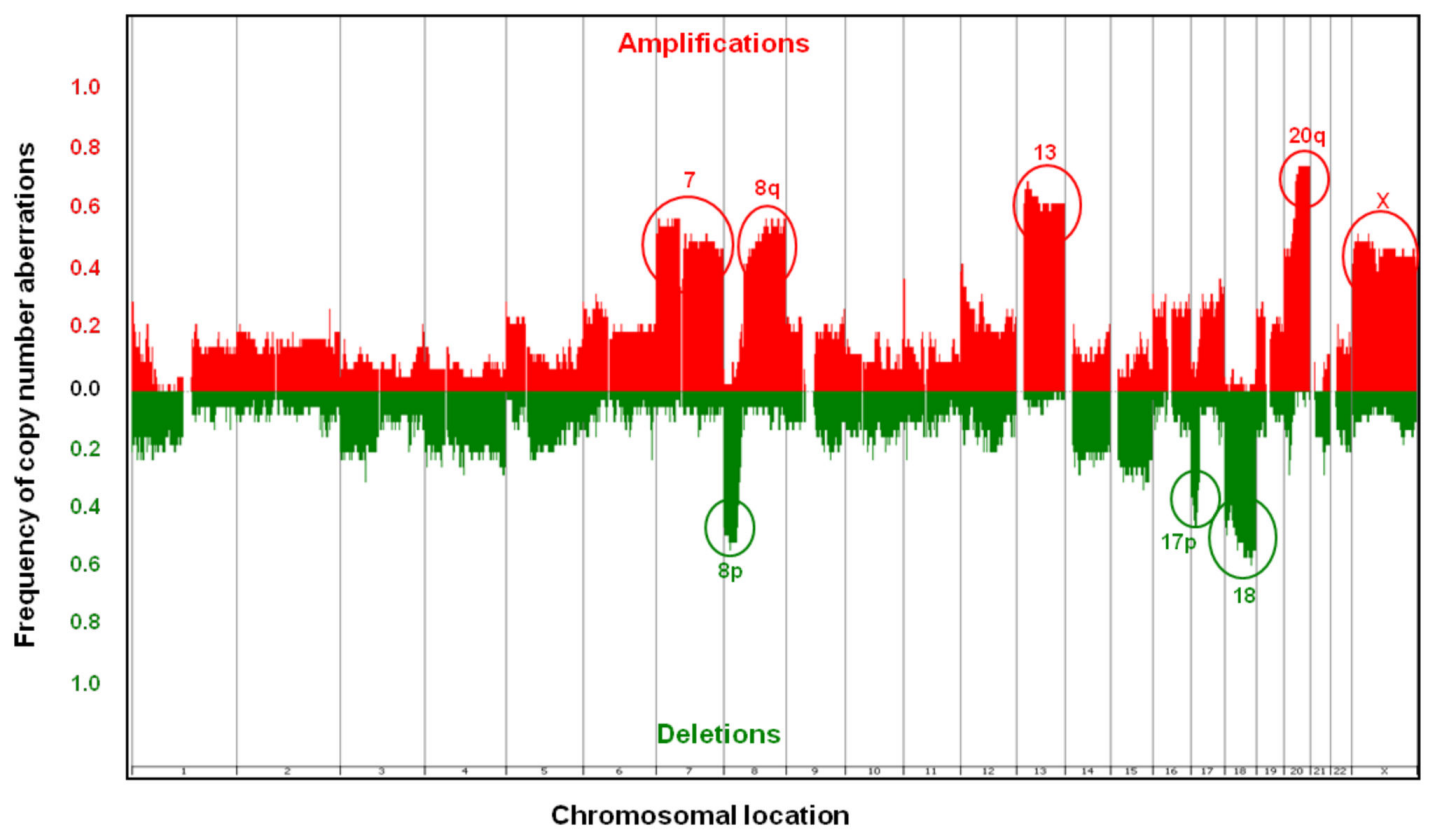
Distribution of CNA in 40 CIN tumour samples. Data from the 180K format array are shown. Red represents gains and green represents deletions. The y-axis reflects frequency.

**Figure 2:**
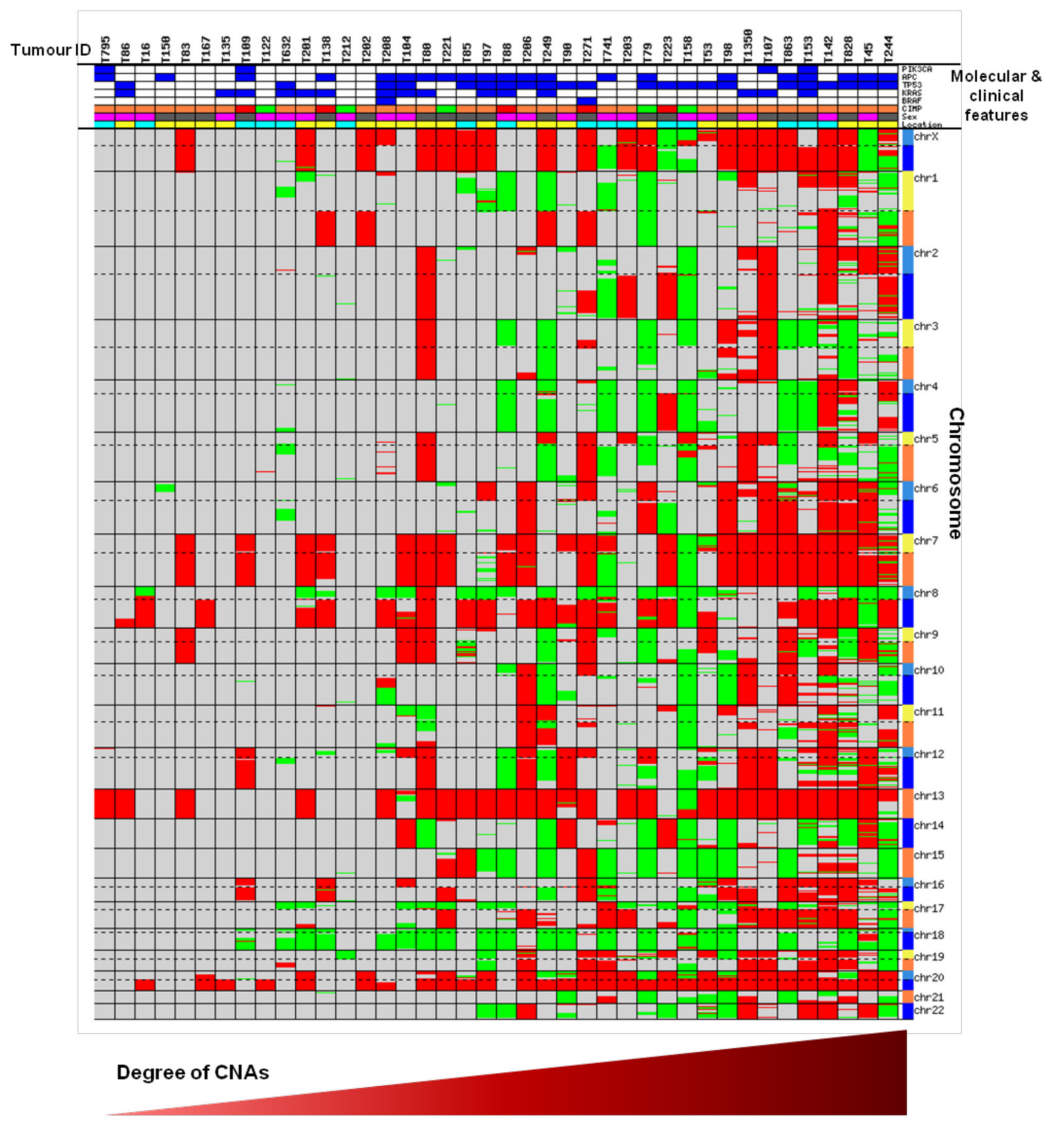
CNA identified in 40 CIN tumour samples (180K format array). Representation of all the CNA identified in 40 chromosomally unstable cases analysed using the 180K platform. Red represents gain and green represents deletion. Dotted lines mark centromeres. Molecular and clinical features in order from top; *PIK3CA*, *APC*, TP53, KRAS and BRAF mutation status (blue and white represents mutant and WT respectively), CIMP (red, orange and green represent CIMP-H, CIMP-L and CIMP-N respectively), patient’s gender (pink and grey represent female and male respectively) and tumour location (yellow and cyan represent proximal and distal respectively).

A total of 3097 SCNA were identified in the 45 chromosomally unstable cases. The number of SCNA per sample ranged from 1-411, with median of 43 per sample. SCNA ranged in size from 0.014Mb-147.48Mb (median: 2.29Mb). An overview of the pattern and frequencies of these SCNA is presented in Figure 1. The most common gains were on chromosome regions 20q (73.3%, n=33), 13 (57.8%, n=26), 8q (53.3%, n=24), 7 (51.1%, n=23) and X (51.1%, n=23) and the most common deletions were carried on chromosome regions 18 (55.6%, n=25), 8p (51.1%, n=23) and 17p (51.1%, n=23). Some of these regions contain key CRC driver genes, such as *MYC* at 8q, *SMAD4* at 18q and *TP53* at 17p. A summary of the SCNA for each sample is presented in Table S1 in File S1 and Figure 2.

### Common aberration analysis and FMCR

Out of the 3097 SCNA identified in the 45 chromosomally unstable samples, 1689 aberrations were focal (<3.0Mb) ranging in size from 0.014Mb up to 3.00Mb (median: 0.71Mb). The focal aberrations consisted of 746 copy gains with a size range of 0.014Mb-2.99Mb (median: 0.92Mb) and 943 deletions, with a size range of 0.025Mb-3.00 Mb (median: 0.58Mb). To identify FMCR, COCA analysis combined with the definitions described in the Methods were applied on the ADM-2 output and this analysis resulted in the identification of 64 deletions and 32 gains meeting the FMCR criteria. The 64 deleted FMCR ranged in size between 0.03-2.64Mb (median: 0.42Mb) and contained a total of 714 known genes with a range of 0-69 genes deleted per region (median: 4 genes) (Table S2 in File S1). The 32 gained FMCR ranged between 0.03-1.96Mb in size (median: 0.83Mb) and contained a total of 288 known genes with a range of 0-34 genes gained per region (median: 3 genes) (Table S3 in File S1). 

### Identification of known cancer genes in the FMCR

To identify known cancer genes within the deleted and gained FMCR, the FMCR gene lists were compared to both complete gene list from the cancer gene census project (http://cancer.sanger.ac.uk/cancergenome/projects/census/, accessed June, 2013), and the CRC and breast cancer driver genes identified in a recent high throughput re-sequencing study [34]. This analysis indicated the presence of 27 “cancer genes” located within the deleted FMCR (~3.8% of the total number of deleted genes; Table S2 in File S1) and 11 “cancer genes” within the gained FMCR (~3.8% of the total number of gained genes; Table S3 in File S1).

The occurrence of a cancer gene within an FMCR does not necessarily imply that it is acting as a driver gene. For some “cancer genes”, the type of FMCR (deleted or amplified) was not consistent with the known gene function, an example being the deletion of the known oncogene *NRAS*. However, the gene function and the type of the FMCR were often consistent with expectation, examples including deletions of *MAP2K4* and *CDKN2C* (Table S2 in File S1) and gain of *FGFR1* (Table S3 in File S1). Perhaps, most importantly, the classical *SMAD4* tumour suppressor deletion and oncogenic *MYC* gain were both observed within deleted and gained FMCR respectively. The frequency of both *SMAD4* deletions and *MYC* gains in the 45 chromosomally unstable cases was high at ~53% (Tables S2 & S3 in File S1).

### Comparison with published SCNA data

In order to cross-validate our results with published data, we compared the FMCR to common chromosomal aberrations of less than 3Mb identified in four recent CRC studies [13,16,22,35]. The total of focal aberrations identified in these studies was 187 gains and 189 deletions. Between the 4 published studies, there were only 10 overlapping focal deletions (5.3%) and 29 overlapping focal gains (15.5%). A higher proportion of the FMCR in our study overlapped the published regions. In total, 27 of our 64 deleted FMCR (42.2%) and 11 of our 32 gained FMCR (34.4%) overlapped focal deletions and gains in the 4 published studies.

A large SCNA study was recently performed in 26 different cancer types, including CRC [17]. The study identified a list of the 20 most common somatic deletions and gains across the analysed cancer types. Moreover, a candidate driver gene was also selected for each of the common SCNA. A comparison between our FMCR and the most common regions in the Beroukhim et al study revealed an overlap with 5 of the deletion areas (25% of total) and 3 of the gain areas (15% of total). All the candidate genes identified by Beroukhim and colleagues in their study were contained within the overlapping areas from our FMCR.

### Pathway analysis

In order to search for any specific patterns or pathways affected by the genes within the deleted or gained FMCR, the Database for Annotation, Visualisation, and Integrated Discovery (DAVID) v6.7 was used (http://david.abcc.ncifcrf.gov/) [36]. DAVID performs enrichment analysis (based on biological annotations for the target genes) to identify any biological pathways that are statistically significantly over-represented in the analysed gene list [36]. For the deleted FMCR, the most enriched cancer-related pathway was Apoptosis (P = 0.014) (Figure S1) with 10 apoptotic genes occurring within the deleted FMCR. Seven of these genes (*CASP3*, *CASP8*, *CASP10*, *NFKBIA*, *CAPN1*, *BAD*, *TNF* and *TNFRSF1A*) have pro-apoptotic roles and 3 (*PIK3R5, PIK3R2 and CFLAR*) are anti-apoptotic. Also, the p53 signalling pathway was enriched in the deleted regions (P = 0.079) with 5 affected genes (Figure S2); *CCNB1*, *GADD45G*, *SERPINE1*, *SESN2* and *SFN*, all of which have reported anti-survival functions. 

On the other hand, the oncogenic MAPK signalling pathway was the most over-represented pathway in the gained FMCR (P = 0.032) with 9 affected genes (Figure S3). Seven of these genes (*CACNA1H*, *FGF23*, *FGF6*, *FGFR1*, *MAPKAPK2*, *ELK4* and *MYC*) are known to have growth promoting and survival functions, while the other 2 (*DUSP8 and DUSP2*) have anti-survival functions.

Gene Relationships Among Implicated Loci (GRAIL) analysis was also performed (http://www.broadinstitute.org/mpg/grail/) [37] to search for functional relationships among the genomic regions. The terms apoptosis, apoptotic and caspase were among the most significantly enriched amongst the deleted FMCR. For the gains, potassium conductance channels and the hedgehog pathways were among the most significantly enriched terms. 

### Identification of candidate driver genes within candidate FMCR

In order to identify novel candidate CRC driver genes, the FMCR criteria were made more stringent. Candidate FMCR were selected if they were defined by 4 SDE, occur in ≥20% of the cases and have a maximum of 12 genes within 3.0Mb of size. These more stringent FMCR definitions identified 11 deletions (Table 1) and 8 gains (Table 2). Out of the 28 genes within the deleted areas, 10 (35.7%) were identified as candidate driver genes with known or potential tumour suppressor functions and out of the 31 genes within the gained regions, 6 (19.4%) were candidates with known or potential oncogenic function (Tables 1 and 2; see Discussion). Candidate tumour suppressor genes in deleted FMCR included *RASSF3*, *IFNAR1*, *IFNAR2 and NFKBIA*, and candidate oncogenes in gained FMCR included *PRDM16, TNS1, RPA3* and *KCNMA1*. Importantly, the copy number status of 2 of these novel candidate driver genes, *NFKBIA* and *KCNMA1*, was confirmed by specific quantitative RT-PCR assays.

**Table 1:** Candidate driver genes in deleted FMCR.

**Chromosomal location**	**Start**	**End**	**Size (Mb)**	**Recurrence (%)**	**SDE**	**Genes**	Candidate genes*
3p14.3	57521404	57652691	0.13	24.44	4	3	
3p14.2	60078018	61195823	1.12	31.11	7	1	*FHIT*
4q22.1	91340468	92674544	1.33	33.33	8	2	*TMSL3*
6q26	162357125	163049854	0.69	22.22	7	1	*PARK2*
11p15.4	9172449	9363610	0.19	22.22	5	3	
12q14.2	63277389	63368109	0.09	20.00	4	1	*RASSF3*
14q13.2	34108596	34950339	0.84	28.89	5	9	*NFKBIA*
16p13.3-p13.2	6132536	7018275	0.89	24.44	7	1	*A2PB1*
17p13.1-p12	10957541	12400968	1.44	48.89	5	3	*MAP2K4*
20p12.1	14376202	16071135	1.69	37.78	10	1	*MACROD2*
21q22.11	33554005	33651205	0.10	28.89	7	3	*IFNAR1, IFNAR2*

^*^ Candidate genes defined based on cancer-relevant functions.

**Table 2:** Candidate driver genes in gained FMCR.

**Chromosomal location**	**Start**	**End**	**Size (Mb)**	**Recurrence (%)**	**SDE**	**Genes**	Candidate genes*
1p36.32	2412144	3630036	1.22	26.67	7	11	*PRDM16*
1p34.3	36881028	37535891	0.65	20.00	5	1	
2q35	218354227	218556081	0.20	20.00	5	1	*TNS1*
7p22.1-p21.3	7033162	7829887	0.80	48.89	5	4	*RPA3*
8q23.3	113827791	114547454	0.72	51.11	4	0	NA
10q22.3	78238542	79084656	0.85	20.00	4	1	*KCNMA1*
12p13.32-p13.31	4282429	5364999	1.08	40.00	4	12	*FGF23, FGF6*
22q11.21	18517833	18686313	0.17	20.00	4	1	

^*^ Candidate genes defined based on cancer-relevant functions.

## Discussion

### Identification of FMCR and affected pathways

The frequency and pattern of mutations in *APC, TP53*, *KRAS*, *BRAF* and *PIK3CA* were typical of MSS/CIN tumours, suggesting that the sample analysed here is representative of this tumour type. FMCR were initially defined as aberrant regions smaller than 3Mb in size, occurring in more than 10% of the cases, with at least 2 SDE and a COCA score ~ ≥2 (p-value = 0.01). Overall, 64 deleted and 32 gained FMCR were identified according to these criteria. The previously published studies showed a rather low level of overlap of aberrations between them (5.3% for focal deletions and 15.5% for focal gains) [13,16,22,35]. This is perhaps not surprising since the use of different platforms, analytical methods and sample sets will result in the identification of different hotspots of aberrations. Our study showed a higher degree of overlap with the previously published data (42.2% of deleted FMCR and 34.4% of gained FMCR), suggesting the absence of any significant levels of bias in our sampling and methods.

Pathway analysis showed that the most significantly enriched cancer-related pathways amongst deleted FMCR were apoptosis and p53 signalling pathways. Ten apoptotic genes were commonly deleted in our samples, 7 of which have known pro-apoptotic functions and 3 (*PIK3R2*, *PIK3R5* and *CFLAR*) have anti-apoptotic roles. Nevertheless, *CFLAR* occurs within the same FMCR as the pro-apoptotic genes *CASP8* and *CASP10*. Similarly, *PIK3R5* is located 1.2Mb downstream of *TP53*, and 81% (n=17) of the deletions are common between the 2 genes. Therefore, it seems likely that it is the deletion of the pro-apoptotic genes within the FMCR which is acting as a driver event, with the anti-apoptotic genes being co-deleted passengers. For the p53 signalling pathway, all the deleted genes (n=5) are known to have anti-tumourigenic activities. It is notable that *TP53* itself was also deleted in 37.8% (n=17) of the cases, but it was not identified within an FMCR. These results suggest that the deleted FMCR might play an important role in tumour cell survival through disabling apoptosis and/or the p53 signalling pathway. Moreover, the enrichment of apoptotic genes within the deleted FMCR supports the known correlation between genomic instability and defective apoptosis [38].

The most significantly enriched pathway for the gained FMCR genes was the oncogenic MAPK pathway, with 9 genes being commonly affected. Seven of these genes are known or predicted to have oncogenic activities by promoting tumour growth and survival. Although, 2 genes have anti-survival roles, one of these (*DUSP8*), occurred within the same FMCR as the oncogenic growth-promoting gene *IGF2*. Overall these results confirm that focal deletions and copy number gains target genes within tumour suppressor and oncogenic pathways respectively.

### Candidate cancer driver genes

In total, the identified FMCR contained ~1000 genes. In order to identify a set of candidate driver genes, the FMCR were further prioritized by use of a more stringent FMCR definition. The shortlisted candidate FMCR were 11 deletions and 8 gains, containing a total of 59 affected genes.

Ten of the 28 genes (35.7%) in the 11 candidate deleted FMCR are cancer-related with known or potential tumour suppressor function. Six of these genes (*FHIT*, *TMSL3*, *PARK2*, *A2BP1*, *MACROD2* and *MAP2K4*) have been consistently reported as deleted in CRC and other cancers [17,22,26,35]. On the other hand, *RASSF3*, *IFNAR1*, *IFNAR2* and *NFKBIA* were not previously reported to be affected in CRC. *RASSF3* was previously shown to inhibit cell proliferation in breast cancer cell lines [39]. Protein levels of interferon receptors genes (*IFNAR1 and 2*) were shown to be down-regulated in bladder cancer, and associated with advanced stage and resistance to chemotherapy [40]. Inactivating mutations of *NFKBIA* have been found in Hodgkin lymphoma, and heterozygous *NFKBIA* deletions were reported in ~25% of GBM cases [23,41].. Based on their functions, the literature and their occurrence within the candidate deleted FMCR, we propose *RASSF3*, *IFNAR1*, *IFNAR2* and *NFKBIA* as novel candidate CRC tumour suppressor genes.

Six of the 31 genes (19.4%) in the 8 candidate gained FMCR are cancer related with predicted oncogenic functions (*PRDM16*, *TNS1*, *RPA3*, *KCNMA1, FGF23* and *FGF6*). *FGF23* and *FGF6* have been reported as gained in CRC and other cancer types [15,17]. However, *PRDM16*, *TNS1*, *RPA3* and *KCNMA1* have not been previously reported in CRC. PR domain-containing 16 gene (*PRDM16*) is a known oncogene implicated in acute myeloid leukaemia (AML) and osteosarcoma [42,43] Tensin 1 gene (*TNS1*) is the only gene present in the relevant FMCR, and *TNS1* overexpression *in vitro* was previously shown to significantly promote cell migration in fibroblasts [44]. Replication protein A3 gene (*RPA3*) was recently shown to be gained in metastatic melanoma (within an FMCR) and to play an essential role in tumour invasion [45]. Large conductance calcium-activated potassium channel alpha subunit gene (*KCNMA1*), the only gene in its FMCR, has been shown to be gained in prostate cancer cases and overexpressed in metastatic breast cancer [46,47]. Based on their functions, the literature and the occurrence within the candidate gained FMCR, we propose *PRDM16*, *TNS1*, *RPA3* and *KCNMA1* as novel candidate CRC oncogenes. 

In summary, our results, based on the analysis of focal minimal common regions, confirm previously reported CRC loci and support the hypothesis that recurrent focal aberrations target cancer-related genes and pathways. Moreover, focal deletions and amplifications were shown to affect known tumour suppressor genes and oncogenes respectively. We propose here several novel candidate CRC driver genes. Further validation and functional studies are required to determine their potential role in CRC tumourigenesis.

## Supporting Information

Figure S1 **Apoptotic genes in the deleted FMCR (DAVID output).** DAVID output showing the apoptosis signalling pathway with deleted genes marked by a red star.
(TIF)

Figure S2 **P53 signalling pathway in the deleted FMCR (DAVID output).** DAVID output showing the P53 signalling pathway, with deleted genes marked by a red star.
(TIF)

Figure S3 **MAPK pathway in the gained FMCR (DAVID output).** DAVID output showing the MAPK signalling pathway with amplified genes marked by a red star.
(TIF)

Materials and Methods S1 **Summary of the patients and tumour characteristics, primers sequences and annealing temperatures and NCBI gene accession numbers.**
(DOC)

File S1 **Tables S1-S3.** Table S1: Summary of the molecular features of the 53 tumour samples. Table S2: Summary of the 64 deleted FMCR. Table S3: Summary of the 32 gained FMCR.
(XLS)
