# Supplementary figures and images for: Identification of Candidate Driver Genes in Common Focal Chromosomal Aberrations of Microsatellite Stable Colorectal Cancer
Source: PLoS One. 2013 Dec 18;8(12):e83859. doi: 10.1371/journal.pone.0083859 (PMC3867468; doi:10.1371/journal.pone.0083859)

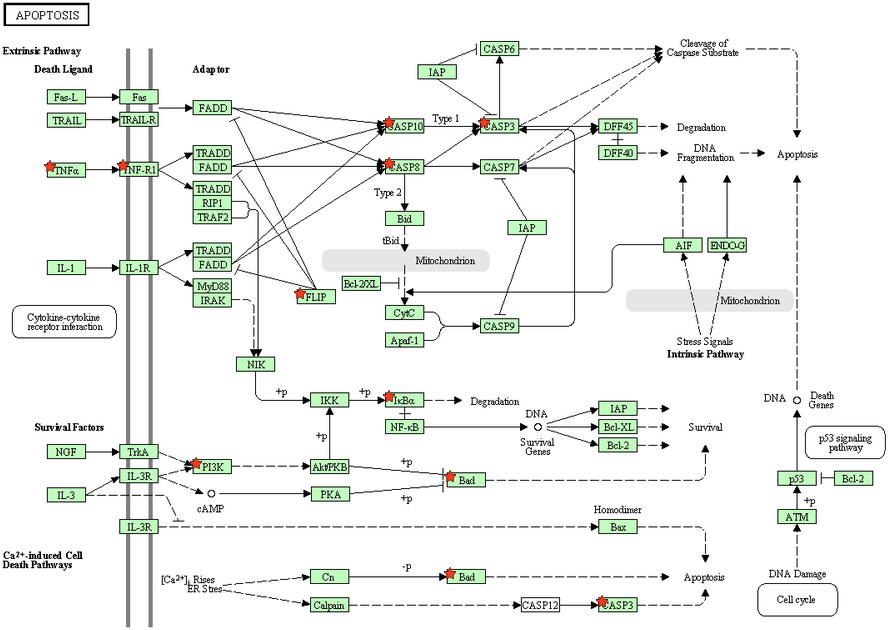

Supplement: Figure S1 — Apoptotic genes in the deleted FMCR (DAVID output). DAVID output showing the apoptosis signalling pathway with deleted genes marked by a red star. (TIF) [file pone.0083859.s001.tif]

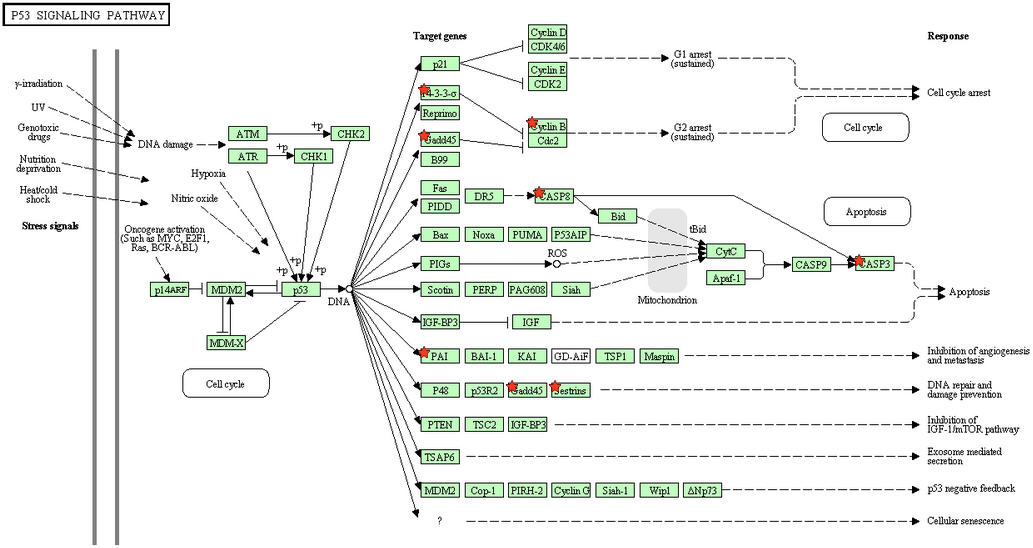

Supplement: Figure S2 — P53 signalling pathway in the deleted FMCR (DAVID output). DAVID output showing the P53 signalling pathway, with deleted genes marked by a red star. (TIF) [file pone.0083859.s002.tif]

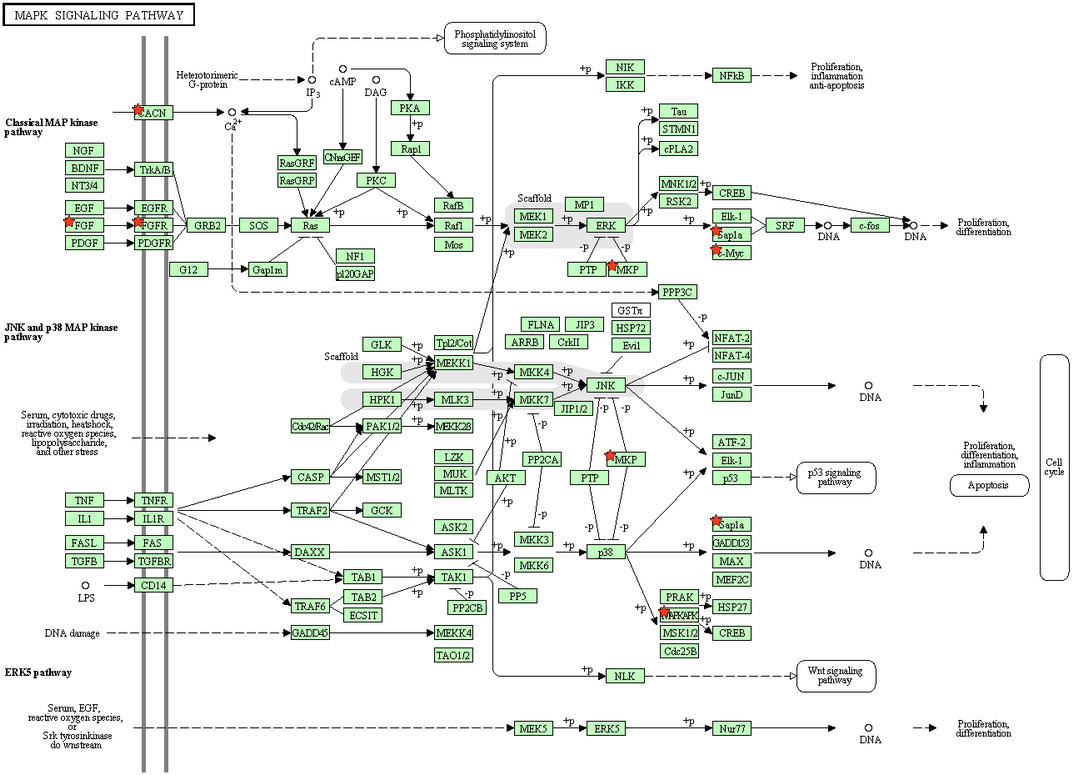

Supplement: Figure S3 — MAPK pathway in the gained FMCR (DAVID output). DAVID output showing the MAPK signalling pathway with amplified genes marked by a red star. (TIF) [file pone.0083859.s003.tif]
