# Supplementary material for: Identification of Candidate Driver Genes in Common Focal Chromosomal Aberrations of Microsatellite Stable Colorectal Cancer
Source: PLoS One. 2013 Dec 18;8(12):e83859. doi: 10.1371/journal.pone.0083859 (PMC3867468; doi:10.1371/journal.pone.0083859)
Supplement: Materials and Methods S1 — Summary of the patients and tumour characteristics, primers sequences and annealing temperatures and NCBI gene accession numbers. (DOC) [file pone.0083859.s004.doc]

Patient and Tumour characteristics

| **Median age (range)** 70.0 (41-90) | | |
| --- | --- | --- |
| **Gender n (%)** | | |
|  | Male | 28 (52.8) |
|  | Female | 25 (47.2) |
| **Tumour location** | | |
|  | Proximal | 19 (35.8) |
|  | Distal | 11 (20.8) |
|  | Rectal | 23 (43.4) |
| **Duke's Stage** | | |
|  | A | 4 (7.5) |
|  | B | 14 (26.4) |
|  | C | 31 (58.5) |
|  | D | 1 (1.9) |
|  | Unknown | 3 (5.7) |
| **Differentiation** | |  |
|  | Well/Moderate | 12 (22.6) |
|  | Moderate | 31 (58.5) |
|  | Poor | 7 (13.2) |
|  | Unknown | 3 (5.7) |

| **Sample ID** | **Tumour Location** | **Sex** | **Age at diagnosis** | **Dukes Stage** | **Differentiation** |
| --- | --- | --- | --- | --- | --- |
| T16 | Proximal | Female | 87 | B | Moderate |
| T23 | Proximal | Male | 79 | C | Moderate |
| T37 | Distal | Male | 57 | A | Moderate |
| T45 | Distal | Female | 80 | C1 | Unknown |
| T46 | Distal | Female | 65 | C1 | Moderate |
| T53 | Distal | Female | 75 | B | Well/Moderate |
| T79 | Distal | Male | 54 | C1 | Well/Moderate |
| T80 | Distal | Male | 64 | B | Well/Moderate |
| T83 | Distal | Male | 74 | NA | Unknown |
| T85 | Proximal | Male | 80 | B | Moderate/Poor |
| T86 | Distal | Female | 65 | C1 | Moderate |
| T88 | Proximal | Female | 70 | C1 | Moderate |
| T90 | Distal | Female | 60 | B | Moderate |
| T97 | Distal | Male | 78 | NA | Well/Moderate |
| T98 | Distal | Male | 81 | B | Well/Moderate |
| T104 | Distal | Female | 76 | C2 | Moderate |
| T107 | Distal | Male | 67 | D | Moderate |
| T109 | Proximal | Male | 56 | C2 | Moderate |
| T110 | Proximal | Male | 78 | C1 | Moderate |
| T112 | Distal | Female | 62 | C1 | Moderate |
| T114 | Distal | Male | 67 | C1 | Poor |
| T122 | Proximal | Female | 90 | C | Moderate |
| T135 | Distal | Female | 87 | B | Moderate |
| T138 | Distal | Male | 77 | C1 | Moderate |
| T142 | Proximal | Female | 58 | C1 | Moderate |
| T150 | Distal | Male | 41 | C | Moderate |
| T153 | Proximal | Male | 83 | C | Poor |
| T158 | Proximal | Female | 76 | NA | Unknown |
| T167 | Distal | Male | 53 | C1 | Moderate |
| T184 | Distal | Male | 72 | C1 | Moderate |
| T201 | Distal | Female | 64 | B | Moderate |
| T202 | Distal | Male | 58 | A | Moderate |
| T203 | Proximal | Female | 87 | B | Well/Moderate |
| T206 | Distal | Female | 81 | B | Well/Moderate |
| T208 | Distal | Female | 51 | C1 | Well/Moderate |
| T212 | Proximal | Female | 82 | C1 | Poor |
| T213 | Distal | Female | 78 | C2 | Well/Moderate |
| T214 | Distal | Male | 53 | B | Well/Moderate |
| T218 | Distal | Female | 52 | C1 | Moderate |
| T221 | Distal | Male | 74 | B | Moderate |
| T223 | Proximal | Male | 75 | C2 | Moderate |
| T244 | Distal | Male | 60 | C1 | Moderate |
| T248 | Distal | Male | 65 | C1 | Moderate |
| T249 | Distal | Male | 65 | B | Moderate |
| T271 | Proximal | Male | 66 | C1 | Well/Moderate |
| T632 | Proximal | Female | 57 | C | Poor |
| T741 | Proximal | Female | 76 | C | Poor |
| T795 | Proximal | Female | 72 | C | Moderate |
| T824 | Distal | Female | 81 | C | Poor |
| T828 | Distal | Male | 70 | A | Moderate |
| T863 | Proximal | Male | 81 | A | Moderate |
| T1120 | Proximal | Male | 66 | C | Well/Moderate |
| T1350 | Distal | Female | 64 | B | Moderate |

***BRAF*** Primers

| **Primer** | **Primer Sequence 5'-3'** | **Length** | **GC%** | **Tm º C** |
| --- | --- | --- | --- | --- |
| *BRAF*-exon15F | CCAGGAGTGCCAAGAGAATATC | 22 | 50.0 | 60.30 |
| *BRAF*-exon15R | AGTAACTCAGCAGCATCTCAGG | 22 | 50.0 | 60.30 |

***KRAS*** Primers

| **Primer** | **Primer Sequence 5'-3'** | **Length** | **GC%** | **Tm º C** |
| --- | --- | --- | --- | --- |
| *KRAS*-exon1F | ACGATACACGTCTGCAGTCAAC | 22 | 50.0 | 60.30 |
| *KRAS*-exon1R | GCACAGAGAGTGAACATCATGG | 22 | 50.0 | 60.30 |

***APC*** Primers

| **Primer** | **Primer Sequence 5'-3'** | **Length** | **GC%** | **Tm º C** |
| --- | --- | --- | --- | --- |
| *APC*MCRf | GTTCTGCACAGAGTAGAAGTGG | 22 | 50.0 | 60.3 |
| *APC*MCRr | GTGATGACTTTGTTGGCATGGC | 22 | 50.0 | 60.3 |
| *APC*MCReqf | CTCCGTTCAGAGTGAACCATG | 21 | 52.4 | 59.8 |
| *APC*MCReqr | CATGGTTCACTCTGAACGGAG | 21 | 52.4 | 59.8 |

***TP53*** Primers

| **Primer** | **Sequence 5'-3'** | **Length** | **GC%** | **Tm ^o^ C** |
| --- | --- | --- | --- | --- |
| *TP53*-exon4F | ACGTTCTGGTAAGGACAAGGG | 21 | 52.4 | 59.8 |
| *TP53*-exon4R | GACAGGAGTCAGAGATCACAC | 21 | 52.4 | 59.8 |
| *TP53*-exon5/6F | AAAGCTCCTGAGGTGTAGACG | 21 | 52.4 | 59.8 |
| *TP53*-exon5/6R | GGGAGGTCAAATAAGCAGCAG | 21 | 52.4 | 59.8 |
| *TP53*-exon7F | AAAAGGCCTCCCCTGCTTGC | 20 | 60.0 | 61.4 |
| *TP53*-exon7R | TGATGAGAGGTGGATGGGTAG | 21 | 52.4 | 59.8 |
| *TP53*-exon8/9F | AGCTTAGGCTCCAGAAAGGAC | 21 | 52.4 | 59.8 |
| *TP53*-exon8/9R | AGTTAGCTACAACCAGGAGCC | 21 | 52.4 | 59.8 |
| *TP53*-exon10F | GTCAGCTGTATAGGTACTTGAAG | 22 | 43.5 | 58.9 |
| *TP53*-exon10R | TGACCATGAAGGCAGGATGAG | 21 | 52.4 | 59.8 |

***PIK3CA*** Primers

| **Primer** | **Primer Sequence 5'-3'** | **Length** | **GC%** | **Tm º C** |
| --- | --- | --- | --- | --- |
| *PIK3CA*exon9F | CAGTTACTATTCTGTGACTGGTG | 23 | 43.5 | 58.9 |
| *PIK3CA*exon9R | TGCTGAGATCAGCCAAATTCAG | 22 | 45.5 | 58.4 |
| *PIK3CA*exon20F | TTGCTCCAAACTGACCAAACTG | 22 | 45.5 | 58.4 |
| *PIK3CA*exon20R | TGCAATTCCTATGCAATCGGTC | 22 | 45.5 | 58.4 |

Genes accession numbers

| **Gene** | **Chromosome** | **Accession number** | **Location** | **Genomic Build** |
| --- | --- | --- | --- | --- |
| *CASP8* | 2q33-q34 | NC_000002.10 | 201804871-201862219 | 36.3 |
| *APC* | 5q21-q22 | NC_000005.8 | 112100455-112210835 | 36.3 |
| *TP53* | 17p13.1 | NC_000017.9 | 7532642-7511445 | 36.3 |
| *KRAS* | 12p12.1 | NG_007524.1 | 5001-50675 | 36.3 |
| *BRAF* | 7q34 | NG_007873.1 | 5001-195753 | 36.3 |
| *PIK3CA* | 3q26.3 | NG_012113.1 | 5001-91190 | 37.1 |
